# Mutation in *Brachypodium* caffeic acid *O*-methyltransferase 6 alters stem and grain lignins and improves straw saccharification without deteriorating grain quality

**DOI:** 10.1093/jxb/erv446

**Published:** 2015-10-03

**Authors:** Séverine Ho-Yue-Kuang, Camille Alvarado, Sébastien Antelme, Brigitte Bouchet, Laurent Cézard, Philippe Le Bris, Frédéric Legée, Alessandra Maia-Grondard, Arata Yoshinaga, Luc Saulnier, Fabienne Guillon, Richard Sibout, Catherine Lapierre, Anne-Laure Chateigner-Boutin

**Affiliations:** ^1^INRA-UR1268 Biopolymères, Interactions, Assemblages, F-44316 Nantes, France; ^2^INRA-UMR1318, Institut Jean-Pierre Bourgin, F-78026 Versailles, France; ^3^Laboratory of Tree Cell Biology, Division of Forest and Biomaterials Science, Graduate School of Agriculture, Kyoto University, Sakyo-ku, Kyoto 606-8502, Japan

**Keywords:** *Brachypodium distachyon*, COMT, ferulic acid, grains, *para*-coumaric acid, lignins, straw saccharification.

## Abstract

Cereal crop by-products are a promising source of renewable raw material for the production of biofuel from lignocellulose. However, their enzymatic conversion to fermentable sugars is detrimentally affected by lignins. Here the characterization of the *Brachypodium Bd5139* mutant provided with a single nucleotide mutation in the caffeic acid *O*-methyltransferase *BdCOMT6* gene is reported. This *BdCOMT6*-deficient mutant displayed a moderately altered lignification in mature stems. The lignin-related *BdCOMT6* gene was also found to be expressed in grains, and the alterations of *Bd5139* grain lignins were found to mirror nicely those evidenced in stem lignins. The *Bd5139* grains displayed similar size and composition to the control. Complementation experiments carried out by introducing the mutated gene into the *AtCOMT1*-deficient *Arabidopsis* mutant demonstrated that the mutated BdCOMT6 protein was still functional. Such a moderate down-regulation of lignin-related COMT enzyme reduced the straw recalcitrance to saccharification, without compromising the vegetative or reproductive development of the plant.

## Highlight

The first evaluation of lignification in *Brachypodium distachyon* grain is reported. Moderately down-regulated BdCOMT6 alters grain and stem lignification, which improves stem saccharification without major detrimental effects on grain development and composition.

## Introduction

Grass lignocellulosics are important potential feedstocks for the production of cellulosic ethanol. Cereal by-products, such as wheat straw or corn stover, and dedicated-energy grass crops, such as miscanthus or switchgrass, are mainly composed of cellulose, hemicellulose, and lignins. However, their enzymatic saccharification into fermentable sugars is detrimentally affected by lignins and their cross-linking to wall carbohydrates. Indeed, lignins limit the accessibility of enzymes to polysaccharides and make necessary the use of costly pre-treatments aimed at improving this accessibility (Yang and Wyman, 2008). Lignins are currently the target of biotechnology with the objective to design plant cell walls more amenable to the saccharification process. Lignin genetic engineering is still more challenging for grass lignins due to the specificities of lignified grass cell walls. While grass lignins are made of guaiacyl (G) and syringyl (S) units together with a lesser amount of *p*-hydroxyphenyl (H) units, like any other angiosperm lignins, their major peculiarity is that *p*-coumaric acid (CA) and ferulic acid (FA) participate substantially in their assembly in the cell wall (Ralph, 2010). FA acylates the arabinose substituents of arabinoxylans, and grass lignins are oxidatively cross-linked to these ferulate esters (Jacquet *et al.*, 1995). CA acylates mainly S lignin units (Ralph, 2010) and to a lower extent arabinose units (Mueller-Harvey *et al.*, 1986). Designing grass cell walls more amenable to bioethanol production requires the use of appropriate model plants provided with the unique specificities of grass lignins. *Brachypodium distachyon* is a plant species whose genomic sequence is released for the Bd21 accession (International Brachypodium Initiative, 2010) and which has recently been championed as a model grass to identify genes important for cereals and energy grasses (Draper *et al.*, 2001; Vogel *et al.*, 2006; Opanowicz *et al.*, 2008; Bevan *et al.*, 2010; Brkljacic *et al.*, 2011; Mur *et al.*, 2011; Rancour *et al.*, 2012). In the challenging context of designing grass cell walls with improved end-use properties, a *B. distachyon* (*Brachypodium*) mutant collection associated with a TILLING (targeting induced local lesion in genome) platform has been recently developed (Dalmais *et al.*, 2013). Using these tools, several *Brachypodium* mutants affected in a lignin-related caffeic acid *O*-methyltransferase (COMT) activity were identified (Dalmais *et al.*, 2013).

This study focused on the *Bd5139* line displaying a single point mutation in the *BdCOMT6* gene to demonstrate that this lignin-related gene (or its homologues in other grasses) is a promising target for lignin genetic engineering. The prospect of a sustainable use of cereal crops will rely on breeding programmes to improve the potential of by-products for bioethanol production while preserving grain quality. The impact of the *Bd5139* mutation on the cell wall phenolics occurring not only in *Brachypodium* lignified stems, the most conventionally studied organ in lignin-deficient mutants or transgenics, but also in *Brachypodium* grains, is reported. This grain study was performed due to the fact that lignins, CA, and FA are thought to have important roles in the conductive and protective tissues of grass grains (Beaugrand *et al.*, 2004; Barron *et al.*, 2007). It was therefore important to evaluate the impact of the lignin-targeted mutation not only on stems, but also on seeds. It is demonstrated that this single mutation substantially affects the cell wall phenolics of both stems and grains, and improves the saccharification of *Brachypodium* mature stems without impairing grain development and composition.

## Materials and methods

### Plant material and growth conditions

The *Bd5139* mutant line mutated in the *BdCOMT6* gene was selected from a *Brachypodium* mutant collection as previously described (Dalmais *et al.*, 2013). *Brachypodium distachyon* L. Beauv. inbred line Bd21-3 were used as control plants. *Brachypodium* plants were grown in 1 litre soil pots in the greenhouse or growth chambers (18h light at 23 °C, then 6h dark at 18 °C photoperiod; 60% relative humidity). Stems were harvested at 40 days after germination (DAG), 60 DAG, or 90 DAG (dried plants). Grains were harvested in batches from at least 30 plants grown in a growth chamber at 11, 21, and 31 d after flowering (DAF) or from dried plants.

Th*e Arabidopsis comt-1* mutant (SALK_002373 line, Alonso *et al.*, 2003) with a T-DNA insertion in AT5G54160 was used for complementation experiments. *Arabidopsis* transformants were selected on Murashige and Skoog (MS) medium with 100 μM hygromycin. For analyses of soluble phenolic compounds, seeds were sown on the same medium without hygromycin. After 4 d at 4 °C, seeds were transferred to continuous light conditions at 21 °C with a light intensity of 110 mE m^–2^ s^–1^ (cool-white fluorescent tungsten tubes; Osram). For the analyses of soluble phenolics, some plantlets were harvested at 5 DAG, weighed, and immediately dropped into a methanol–water mixture (80/20, v/v). The methanol suspensions were then stored at –80 °C before analyses. Inflorescence stems were harvested at full maturity.

### Cloning of the *BdCOMT6* coding sequence and mutagenesis

Stems from 60 DAG Bd21-3 plants were snap-frozen and ground in liquid nitrogen. Total RNA was extracted using the EZ-10 Total RNA Miniprep Kit (Bio Basic). Contaminant DNA was removed using an RNase-free DNase set (Qiagen), and DNA-free RNA was concentrated using the RNeasy MinElute Cleanup Kit (Qiagen) following the manufacturer’s instructions. RNA (0.25 μg) was reverse transcribed using the Transcriptor First strand cDNA Synthesis kit (Roche) with random hexamers in a 20 μl reaction volume.

The wild-type (WT) *BdCOMT6* (Bradi3g16530) full-length coding sequence was amplified from 1.5 μl of cDNA in a total of 25 μl with the primers BdCOMT6Attb1_ATG 5′GGGGACAAGTTTGTACAAAAAAGCAGGCTCCA TGGGTTCCACGGCGGCGGACAT3′ and BdCOMT6Attb2_stop 5′GGGGACCACTTTGTACAAGAAAGCTGGGTCCTACT TGGTGAACTCGATGGCCC3′ using the KOD Hot Start DNA polymerase (Novagen) and cloned into the vector pDONR207 using Gateway^©^ technology (Life Technology). The QuickChange II Site-Directed Mutagenesis Kit (Agilent) was used to introduce the G767A point mutation corresponding to the mutation in the *Bd5139* line following the manufacturer’s instructions and using the primers BdCOMT6mut_fw 5′CCAGAAGGTCCCCTC GGACGATGCCATCC3′ and BdCOMT6mut_rev 5′GGATGGCAT CGTCCGAGGGGACCTTCTGG3′. The mutated *BdCOMT6* gene (*BdCOMT6^5139^*) was transferred into the pIPKb2 vector containing the maize ubiquitin promoter (Himmelbach *et al.*, 2007). The recombined plasmid was checked by sequencing.

### Genetic complementation of an *AtCOMT1*-deficient *Arabidopsis* mutant with the mutated *BdCOMT6* gene

The T-DNA-insertion *comt-1* mutant available from the SALK collection (SALK_002373) and in the Columbia (Col-0) background was used for complementation assays with the *BdCOMT6* ^*5139*^ gene according to a previously published procedure (Bouvier d’Yvoire *et al.*, 2013). pIPKb2 containing *BdCOMT6* ^*5139*^ was introduced into the *Agrobacterium tumefaciens* C58pMP90 strain (Koncz and Schell, 1986) and *comt-1* plants were infiltrated as described in Zhang *et al.* (2006). Transgenic plants were selected on MS medium containing 50mg l^–1^ hygromycin.

### Analyses of cell wall phenolics from stem or grain samples

The stem or grain samples ground to 0.5mm were exhaustively extracted by cold water, then 60 °C ethanol before freeze-drying to obtain a cell wall residue (CWR).

The Klason lignin (KL) content of stem CWR was measured by the Klason method as previously described (Méchin *et al.*, 2014). The lignin content of grain CWR, available in a lower amount, was measured by the spectrophotometric acetyl bromide method as follows. About 10mg of dry CWR were put in a 2ml glass screw-capped reaction vial (Teflon lined) together with 1ml of freshly made 25% (v/v) acetyl bromide/glacial acetic acid mixture. A blank vial was included which contained the digestion reagent without any sample. The reaction vials were incubated at 55 °C for 2.5h in an Eppendorf Thermomixer and with stirring at 500rpm. The reaction mixture was then cooled and 200 μl of the clear supernatant was diluted with 3ml of 50/9 (v/v) acetic acid/2M NaOH mixture and 500 μl of 0.5M aqueous hydroxylamine hydrochloride solution. The UV spectrum of the diluted solution was registered between 400nm and 250nm and against a blank cell prepared by a similar dilution performed on the blank vial reaction mixture. The acetyl bromide lignin (ABL) concentration was calculated from the absorbance peak at 280nm and using an extinction coefficient value of 20g^–1^ l cm^–1^.

The lignin structure of stem CWR was investigated using the simplified thioacidolysis protocol, as previously described (Méchin *et al.* 2014). The lignin structure of grain CWR was studied in a similar way, but using a modified thioacidolysis reagent made from a 9/1 dioxane/ethanethiol mixture containing 0.1M tetrafluoroboric acid dimethylether complex (Sigma-Aldrich) as previously described (Greffeuille *et al.*, 2006). The specific lignin-derived thioacidolysis monomers were analysed by GC-MS of their trimethylsilylated (TMS) derivatives (Méchin *et al.*, 2014). The determination of the H, G, S, and 5-hydroxyguaiacyl (5-OH G) TMS derivatives was carried out on ion chromatograms reconstructed, respectively, at *m/z* 239, 269, 299, and 357, as compared with the internal standard C21 hydrocarbon evaluated on the ion chromatogram reconstructed at *m/z* (57+71+85).

The analysis of CA and FA ester-linked to the cell walls was carried out by mild alkaline hydrolysis performed from ~10mg of stem or grain samples together with 1ml of 1M NaOH and 0.1mg (for stem CWR) or 0.05mg (for grain CWR) of *o*-coumaric acid internal standard overnight and on a carousel. The reaction mixture was then acidified with 0.2ml of 6M HCl. After centrifugation (1500 *g*, 10min), ~0.5ml of the supernatant was deposited onto a solid-phase extraction cartridge (Waters Sep-pack t18) pre-conditioned before use (washed with 2×3ml of MeOH, then with 2×3ml of water containing 0.01% HCOOH). The sample-loaded cartridge was washed with acidified water and eluted with 2ml of HPLC-grade methanol. The recovered methanolic sample was stored for 30min at –20 °C for complete precipitation of insoluble components before ultrafiltration (0.45 μm) and analysis by HPLC combined with diode array detection (HPLC-DAD). For HPLC separation, 1 μl of sample was injected onto an RP18 column (4×50mm, 2.7 μm particle size, Nucleoshell, Macherey-Nagel) with a flow rate of 0.5ml min^–1^. The eluents were 0.1% formic acid in water (A) and 0.1% formic acid in acetonitrile (B), and the gradient was as follows: 0–3min, 0% B; 12min, 20% B; 14min, 80% B; 16min, 0% B. The quantitative determination of alkali-released CA and FA was performed from the 250–400nm DAD chromatograms and after calibration with authentic compounds.

In addition to the analysis of CA and FA ester-linked to the cell walls, the determination of *p*-coumaroylated arabinose (CA-Ara) and of feruloylated arabinose (FA-ara) units occurring in cell wall arabinoxylans was performed according to a recently developed mild acidolysis procedure (Petrik *et al.*, 2014).

### Analyses of soluble phenolics from *Arabidopsis* plantlets

The LC-MS analyses of soluble phenolics extracted from 5-day old *Arabidopsis* plantlets was carried out as previously described (Do *et al.*, 2007).

### Saccharification assays of mature stem samples

The CWRs obtained from dry stems were subjected to a saccharification assay. About 30mg of stem CWR was put into a 5ml disposable plastic tube together with 4ml of 5mM acetate buffer (pH 4.7) containing 4mg ml^–1^ of a commercial cellulase preparation (cellulase Onozuka-R-10 from *Trichoderma viride*, Serva) and 0.1mg ml^–1^ NaN_3_. A blank tube was prepared containing the enzyme solution without any CWR. The reaction tubes (biological triplicates for each genotype plus the blank tube) were placed at 45 °C for 72h and on a carousel. After centrifugation (1500 *g*, 10min), the supernatant was subjected to glucose determination as follows. In a disposable 1ml spectrophotometric cuvette, 50 μl of the supernatant were mixed together with 1ml of Biomerieux reagent (Biomerieux Glucose RTU kit, Biomerieux, Lyon, France) and the colorimetric reaction was allowed to proceed for 30min. The absorbance was read at 505nm (against a cuvette containing 50 μl of H_2_O and 1ml of reagent) and corrected for the glucose from the blank assay. The amount of glucose released by enzymatic hydrolysis of the stem CWR was calculated after appropriate calibration with standard glucose solutions. The pellets obtained from the CWR subjected to cellulase treatment were washed twice with water (with centrifugation for 10min at 1500 *g* between each wash) before freeze-drying and weight determination.

### Polysaccharide analysis in grain samples

The mature grain polysaccharide content and composition were analysed after recovering the alcohol-insoluble residue (AIR) containing cell wall polysaccharides and starch. The AIR was obtained as described in Guillon *et al.* (2011). Polysaccharide content and composition were determined following their hydrolysis by monitoring the individual neutral sugar content as described in Dervilly *et al.* (2000) according to the method of Englyst and Cummings (1988).

### Histochemistry analyses

Pieces of *Brachypodium* and *Arabidopsis* stems were embedded in 8% agarose, cut into 100 μm sections, and stained using the Maüle and the Wiesner (phloroglucinol-HCl) reagents as described in Bouvier d’Yvoire *et al.* (2013). Cross-sections were observed using a Leica DMRB microscope equipped with a Progress C10plus camera.

To facilitate sectioning, dry grains were placed onto moist paper for 24h (the embryo was removed prior to imbibition).

Grains were cut with a cryotome into 20 μm thick sections to visualize the structure or into 50 μm thick sections for staining using the Maüle or the Wiesner reagents. Grain sections were stained as described for stem in Bouvier d’Yvoire *et al.* (2013). Observations were carried out using a Multizoom macroscope (AZ100M, NIKON). Grains were fixed overnight in 3% (w/v) paraformaldehyde and 0.5% (w/v) glutaraldehyde in 0.1M Na-phosphate-buffered saline (PBS) pH 7.2. Fixed samples were washed, impregnated, and embedded with London Resin White acrylic as described in Chateigner-Boutin *et al.* (2014). The fixed samples were cut into semi-thin sections (1 μm) with an ultramicrotome (UC7, Leica) and used for differential interference contrast (DIC) imaging, toluidine blue staining, and immunolabelling. Sections were directly observed with a microscope (DMRB, Leica) equipped with standard DIC optics using a Plan-APO ×20 objective and Nikon DS-1QM camera. Toluidine blue staining was used to better visualize the testa cell layers. The sections were stained with 0.1% (w/v) toluidine blue O as described in Chateigner-Boutin *et al.* (2014) before observation with a microscope (Axiovert 135, Zeiss) equipped with a QImaging Retiga 2000R Scientific CCD Camera.

For immunolabelling, the semi-thin sections were incubated in a blocking buffer [1% BSA, 0.1% NaN_3_ in Tris-buffered saline (TBS) pH 8.2] for 1h at room temperature. Thereafter, sections were incubated in KM1 ascites fluid (Kiyoto *et al.*, 2013; 1:100 dilution in blocking buffer) for 2 d at 4 °C. After three 5min washes with TBS, sections were incubated with goat anti-mouse IgG Alexa Fluor 568 (Invitrogen, USA, 1:50 dilution in blocking buffer) for 4h at 35 °C After three 5min washes with TBS, sections were water-washed, dried, and then observed under a fluorescence microscope (BX50, Olympus Japan) equipped with a filter set (Semrock TxRed-4040C, Opto-Line Inc., Tokyo, Japan) and a CCD camera (DP72, Olympus Japan). A control experiment was conducted omitting the primary antibody to check for autofluorescence and non-specific labelling.

## Results and Discussion

### The lignin-related *BdCOMT6* gene is expressed both in stem and in grain

In a recent study (Dalmais *et al.*, 2013), *Brachypodium* mutants for the *BdCOMT6* gene were identified in a sodium azide-induced mutant collection established in the Bd21-3 genetic background. Several mutant lines displayed a lower lignin content in mature stems together with a reduced frequency of S lignin units, and *Bd5139* was the most affected line (Dalmais *et al.*, 2013). These results established that the *BdCOMT6* gene is involved in lignification of *Brachypodium* stems.

To characterize the *BdCOMT6* gene further, its expression pattern was obtained using the online expression platform PlaNet/Brachypodium (http://aranet.mpimp-golm.mpg.de/; Mutwil *et al.*, 2011). This investigation revealed a wide expression pattern of the *BdCOMT6* gene. It was found to be expressed not only in vegetative lignified organs, such as nodes and internodes, but also in grains (Supplementary Fig. S1 available at *JXB* online). The *BdCOMT6* expression level was found to be similar in the WT and the *BdCOMT6*-deficient *Bd5139* lines (Supplementary Fig. S1).

### The *Bd5139* mutant has a normal growth phenotype, but an altered lignification in stem and grain

**Fig. 1.**
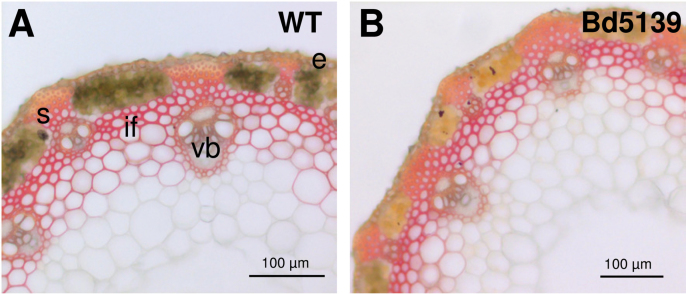
Maüle staining of stem cross-sections of *Brachypodium* WT (Bd21-3) and *Bd5139* lines. *Brachypodium* Bd21-3 (WT; A) and Bd5139 (B) stem cross-sections from 40-day-old plants were stained with the Maüle reagent that stains S lignin units. e, epiderm; if, interfascicular fibres, s, sclerenchyma; vb, vascular bundle. (This figure is available in colour at *JXB* online.)

As compared with the WT line, the *Bd5139* mutant did not show any visible growth or developmental phenotype when grown in a growth chamber or in a greenhouse even though stem biomass was found to be slightly reduced (Supplementary Fig. S2 at *JXB* online). In 40-day-old plants, Maüle staining revealed the occurrence of S lignin units (Nakano and Meshitsuka, 1992) in the cell walls of several stem tissues (epidermis, sclerenchyma, and vascular bundles) without any noticeable difference between the WT and the *Bd5139* lines (Fig. 1). This result suggests that the *Bd5139* mutation might not affect the frequency of S lignin units to a large extent.

It is now well established that lignin-related COMT enzymes of angiosperm species catalyse the 5-OH group methylation on the pathway towards sinapyl alcohol, the precursor of S lignin units (Bonawitz and Chapple, 2010; Vanholme *et al.*, 2010). According to several studies, their most likely *in vivo* substrates would be 5-hydroxyconiferaldehyde and 5-hydroxyconiferyl alcohol (Zubieta *et al.*, 2002; Louie *et al.*, 2010; Green *et al.*, 2014). In contrast, caffeic acid, which has been shown to be an *in vitro* COMT substrate converted into FA (Higuchi, 2006), would be a poor *in vivo* candidate (Parvathi *et al.*, 2001; Green *et al.*, 2014). Manipulation of lignin in plants has repeatedly been carried out by targeting the lignin-related COMT genes, which most often resulted in a deficit of S lignin units and the appearance of unusual levels of 5-OH G units (reviewed in Rastogi and Dwivedi, 2008). In agreement with a preliminary study on COMT-deficient *Brachypodium* lines (Dalmais *et al.*, 2013), both lignin content and lignin structure were found to be affected by the single Gly256Asp mutation occurring in the *Bd5139* line (Table 1). Relative to the WT level, the lignin content of *Bd5139* stem CWR was moderately reduced by ~10%. Lignin structure was more markedly affected by the *BdCOMT6* mutation, as revealed by thioacidolysis (Table 1). This method provides specific monomers from lignin units that are only involved in labile β-O-4 bonds. When expressed relative to the KL content, the total yield of thioacidolysis monomers released from *Bd5139* lignins was found to be lower than the WT value. This reduced yield is diagnostic for an increased frequency of resistant interunit bonds in lignins. In addition, the relative frequency of S thioacidolysis monomers was reduced by the mutation whereas 5-OH G monomers were recovered in an unusually high amount. As previously described in the maize *bm3* mutant (Lapierre *et al.*, 1988; Barrière *et al.*, 2004), sorghum *bmr12* mutant (Palmer *et al.*, 2008), *Arabidopsis Atomt1* mutant (Goujon *et al.*, 2003), COMT1-silenced transgenic tobacco (Pinçon *et al.*, 2001), or COMT-deficient transgenic poplars (Lapierre *et al.*, 1999; Jouanin *et al.*, 2000), these alterations in lignification are the hallmarks of lignin-related COMT deficiency in angiosperms, the most diagnostic signature being the increased frequency of lignin 5-OH G units. While these 5-OH G units were found to build up substantially in the lignins of *Bd5139* stems (up to 5% of the total amount of thioacidolysis monomers), the frequency of S thioacidolysis monomers was found to be only moderately reduced (from 66% in the WT to 57% in *Bd5139*; Table 1). This result is consistent with the observation that the Maüle stain, specific for S lignin units, was not affected to a large extent in *Bd5139* mature stem cross-sections (Fig. 1) in contrast to the observations made for severe *comt* mutants (Jouanin *et al.*, 2000; Pinçon *et al.*, 2001). Taken together, these data reveal that the biosynthesis of sinapyl alcohol is affected to a noticeable but limited extent in the *Bd5139* stems.

**Table 1.** *Lignin content and structure of cell wall residues prepared from wild-type (WT, accession Bd21-3) and* Bd5139 *3-month-old mature stems*Lignin content is evaluated as the Klason lignin (KL) level and lignin structure is evaluated by thioacidolysis.

Line	% KL	Main H, G, S, and 5-OH G thioacidolysis monomers (total yield and relative mol%)				
		Yield (μmol g^–1^ KL)	%H	%G	%S	%5-OH G
WT	19.57±0.30	1258±30	3.1±0.2	29.3±1.2	66.9±1.1	0.8±0.0
*Bd5139*	17.66±0.93*	888±31**	2.9±0.2	36.4±1.2**	55.6±1.5**	5.1±0.4**

Values are means ±SD from three different plants.

The KL level is expressed as weight percentage of the stem cell wall residue.

Asterisks indicate significant differences (*t*-test) compared with the WT value at *P<0.05 or **P<0.01

**Fig. 2.**
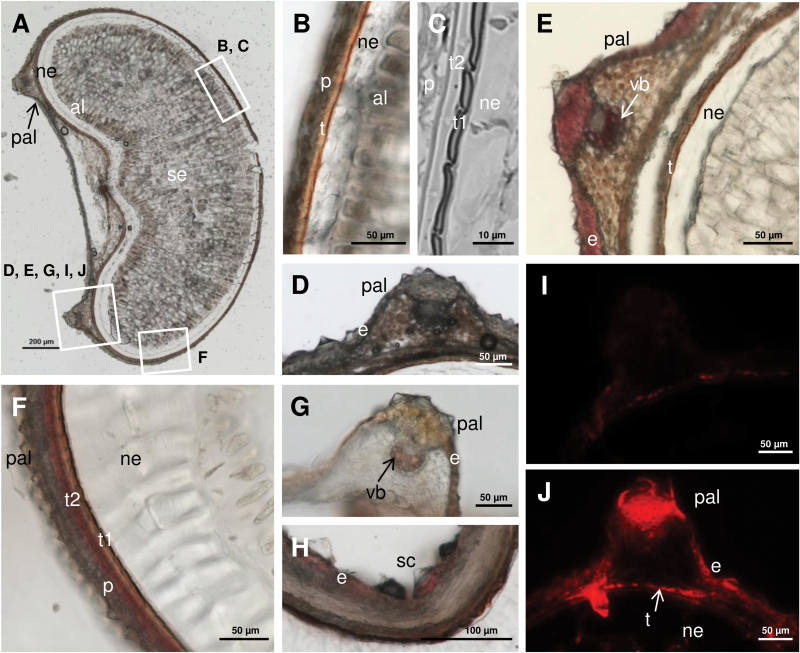
Cytological observations of *Brachypodium* grain. *Brachypodium* Bd21-3 (WT) mature grain cross-sections. (A) Unstained sections of a whole grain with labelled frames indicating the corresponding areas in the subsequent parts of the figure. (B) Unstained section and (C) section stained with toluidine blue focusing on the testa area to visualize the two layers of the testa t1 and t2. (D) Unstained section focusing on one vascular bundle of the grain palea. (E) Section stained with phloroglucinol-HCl revealing positive staining in the palea epidermis and vascular bundle. (F–H) Section stained with Maüle reagent revealing positive staining in the testa outer layer t2, in the palea epidermis, and in the vascular bundle. (J) Section labelled with KM1, an antibody targeting a lignin β-5 structure and showing a positive signal in the testa and in the palea epidermis and vascular bundle, to be compared with (I) the corresponding control without primary antibody. al, aleurone; e, epiderm; ne, nucellar epidermis; p, pericarp; pal, palea; sc, silica cells; se, storage endosperm; t. testa; t1, testa inner layer pigmented; t2, testa outer layer not pigmented but lignified; vb, vascular bundle. (This figure is available in colour at *JXB* online.)

As *BdCOMT6* was found to be expressed in grains and as it is now well established that lignins occur in the outer layers of grass grains (Desvignes *et al.*, 2006; Grefeuille *et al.*, 2006), the lignins from *Brachypodium* grains were studied. The occurrence and distribution of lignins in grain tissues were first studied on grain cross-sections using the Wiesner and the Maüle tests, two commonly used stains specific for lignified tissues. The Wiesner test stains the *p*-hydroxycinnamaldehyde end-groups occurring in native lignins (mainly as coniferaldehyde end-groups) reddish-purple while the Maüle test stains S lignin units deep-purple (Nakano and Meshitsuka, 1992). *Brachypodium* grains are hulled grains consisting of the caryopsis surrounded by two husks, a large lemma, and a small palea (Evers and Millar 2002; Guillon *et al.*, 2011). The lemma can be easily removed while the palea adheres to the pericarp in the crease. In the palea, both the Wiesner and the Maüle tests positively stained the epidermis and vascular bundles (Fig. 2E, G, H), which establishes the occurrence of lignin coniferaldehyde end-groups and of S lignin units in this protective husk. In addition, the outer layer of the seed testa (t2 in Fig. 2C, F) was positively stained by the Maüle reagent whereas no positive Maüle staining could be detected in the brownish-pigmented t1 layer. The Maüle-stained t2 layer did not positively react to the Wiesner test, a phenomenon probably accounted for by the distinct detection sensitivity level of these lignin staining methods. Consistently with the positive Wiesner and Maüle stainings of the seed palea and t2 testa, immunolabelling experiments carried out with an antibody targeting a β-5 dimer of coniferyl alcohol (Kiyoto *et al.*, 2013) confirmed the occurrence of lignins in the grain palea and testa (Fig. 2J).

To evaluate the impact of BdCOMT6 deficiency on the grain lignin level, the WT and *Bd5139* grain CWRs were subjected to ABL determination. The ABL content was found to be similar in the *Bd5139* and WT grain samples (Table 2), in contrast to the mature stem samples (Table 1). CWR samples prepared from whole grain were then subjected to thioacidolysis with the objective of finding a more diagnostic lignin signature. In agreement with previous studies performed on wheat grains (Desvignes *et al.*, 2006; Grefeuille *et al.*, 2006), thioacidolysis unambiguously revealed the occurrence of H, G, and S lignin units in *Brachypodium* whole grains from the detection of H, G, and S thioacidolysis monomers (Table 2). The relative frequency of H thioacidolysis monomers was found to be higher from grain CWR compared with the levels observed from stem CWR. In addition, the relative frequency of S thioacidolysis monomers was found to be substantial (60% of thioacidolysis monomers for the WT sample; Table 2), in agreement with the Maüle positive staining of the palea and of the outer t2 testa layer. More importantly and relative to the WT sample, lignins of *Bd5139* grain displayed severe structural alterations, namely a reduced frequency of S monomers together with an increased frequency of 5-OH G monomers (up to 10% of the thioacidolysis monomers; Table 2; Supplementary Fig. S3 at *JXB* online). When expressed relative to the ABL content, thioacidolysis yield was found to be reduced by the *Bd5139* mutation, which reveals that *Bd5139* grain lignins are richer in resistant interunit bonds than those of the WT. Taken together, the present results establish that the alterations induced by the *Bd5139* mutation in the lignins of mature grains mirror those observed in mature stems. In both samples and relative to the WT, marked lignin structural alterations can be observed, namely a lower frequency of S units together with an increased frequency of 5-OH G units and of resistant interunit bonds. In both *Bd5139* stem and grain samples and surprisingly enough, the frequency S units was found to be reduced, but only to a moderate extent. This persistence of substantial levels of S units in *Bd5139* lignins suggests that some enzyme activity survives in the mutated BdCOMT6 protein.

**Table 2.** *Lignin content and structure of cell wall residues prepared from wild-type (WT, accession Bd21-3) and* Bd5139 *whole grain samples*Lignin content is evaluated as the acetyl bromide lignin (ABL) level and lignin structure is evaluated by thioacidolysis.

Line	% ABL	Main H, G, S, and 5-OH G thioacidolysis monomers (total yield and relative mol%)				
		Yield (μmol g^–1^ ABL)	%H	%G	%S	%5-OH G
WT	3.66±0.50	208±27	5.9±0.1	33.8±2.7	60.2±2.7	Trace
*Bd5139*	3.62±0.15	115±31**	6.2±0.6	39.5±1.8**	44.1±2.4**	10.1±2.6**

Whole grain samples correspond to the whole caryopsis with the adhering palea.

Four biological replicates were prepared for each genotype, with 70–100 grains collected per replicate.

Values are means ±SD.

The ABL level is expressed as weight percentage of the sample cell wall residue.

Asterisks indicate significant differences (*t*-test) compared with the WT value at **P<0.01.

### Evaluation of *p*-coumaric and ferulic acid linked to *Brachypodium* stem and grain cell walls

It is now well established that CA and FA are linked to the polymers of grass cell walls (reviewed in Ralph, 2010). For many grass species, such as maize, sorghum, miscanthus, or wheat, CA and FA units are quite distinctly distributed between the wall polymers of lignified stems. While FA predominantly acylates the arabinose substituents of arabinoxylans, CA is primarily ester-linked to S lignin units. However, such a distinct distribution is not as clearly observed in *Brachypodium* cell walls, which contain a substantial proportion of CA acylating arabinoxylans (Christensen *et al.*, 2010; Petrik *et al.*, 2014).

The effect of the *Bd5139* mutation on the amount of CA or FA ester-linked to the cell wall polymers was monitored by mild alkaline hydrolysis (Table 3). Mature stem cell walls were found to contain higher amounts of CA and FA esters than mature grain cell walls. This compositional trait reflects the higher abundance of lignins and arabinoxylans in stems than in grains, as lignins and arabinoxylans, respectively, are the major *p*-coumaroylated and feruloylated wall polymers.

**Table 3.** *Determination of* p*-coumaric acid (CA) and of ferulic acid (FA) released by mild alkaline hydrolysis (NaOH 1M, room temperature, overnight) of wild-type (WT, accession Bd21-3) and* Bd5139 *cell wall residues (CWRs) prepared from mature stem or whole grain samples*

Line	Mature stem CWR		Mature whole grain CWR	
	CA (mg g^–1^)	FA (mg g^–1^)	CA (mg g^–1^)	FA (mg g^–1^)
WT	8.88±0.41	5.25±0.48	0.98±0.1	1.83±0.47
*Bd5139*	6.30±0.66*	5.33±0.17	0.59±0.04*	1.55±0.11*

Whole grain samples correspond to the whole caryopsis with the adhering palea.

Four biological replicates were prepared for each genotype, with 70–100 grains collected per replicate.

Values are means ±SD (*n*=3).

Asterisks indicate significant differences (*t*-test) compared with the WT value at *P<0.05.

Relative to the WT level, the amount of FA released by mild alkaline hydrolysis was not affected in the *Bd5139* stem CWR and slightly decreased in the mutant grain CWR. This result suggests that BdCOMT6 deficiency has no or little effect on the level of FA released by mild alkaline hydrolysis of *Brachypodium* cell walls. In contrast, mature internodes of COMT-deficient *bm3* maize (Provan *et al.*, 1997; Marita *et al.*, 2003; Barrière *et al.*, 2004) or *bmr12* sorghum (Palmer *et al.*, 2008) lines have been shown to release more FA when subjected to mild alkaline hydrolysis, a phenomenon related to their markedly lower lignin level as measurable FA is reduced by lignification (Grabber *et al.*, 2004). These results confirm that *in planta* the lignin-related COMT activity is not involved in the synthesis of FA that subsequently acylates arabinoxylans.

Relative to WT values, the content of CA esters was reduced by 30% and 40% in *Bd5139* stem and grain samples, respectively (Table 3). A similar reduction of CA units ester-linked to stem cell walls has been reported for maize *bm3* and sorghum *bmr12* mutant lines (Barrière *et al.*, 2004; Palmer *et al.*, 2008). On the rationale that CA mainly acylates maize or sorghum S lignin units, the CA reduction observed in these COMT-deficient lines could be directly assigned to the COMT deficiency-induced reduction of S lignin units. In contrast to maize or sorghum cell walls, a noticeable amount of CA units acylates the arabinoxylans of *Brachypodium* cell walls. It was therefore necessary to clarify whether the lower level of CA esters in *Bd5139* samples affected only lignins or both lignins and arabinoxylans. To address the issue of CA acylation targets, a recently developed mild acidolysis method which efficiently releases CA-Ara and FA-Ara from grass arabinoxylans was employed (Petrik *et al.*, 2014). The CA-Ara quantities released from *Bd5139* stem CWR were found to be very close to the WT values (Supplementary Table S1 at *JXB* online). On this basis, it could be ascertained that the *BdCOMT6* mutation specifically reduces lignin *p*-coumaroylation in *Bd5139* CWR whereas the *p*-coumaroylation of arabinoxylans is not affected. Similar to what was reported in COMT-deficient *bm3* maize or *bmr12* sorghum lines, the *BdCOMT6* mutation affects the biosynthesis of sinapyl alcohol and thereby of sinapyl *p*-coumarate, the precursor of *p*-coumaroylated S lignin units.

### Complementation experiments reveal that the mutated BdCOMT6 protein is still functional in the *Bd5139* mutant

The mutation in *Bd5139* changed a relatively conserved Gly256 residue into an aspartic acid residue (Supplementary Fig. S4 at *JXB* online). The overall impact of this single mutation in the BdCOMT6 protein was found to mimic the impact of the *bm3* or *bmr12* mutations on the lignification of maize or sorghum mutant lines, respectively, but to a less severe extent. Indeed, S thioacidolysis monomers accounted for ~56% of the total amount of thioacidolysis monomers recovered from stem *Bd5139* lignins (versus 67% for the WT; Table 1). In contrast, their frequency was reduced to a value close to zero in the lignins of a *bmr12* sorghum mutant (Palmer *et al.*, 2008) and to a twice lower value relative to the WT level in *bm3* lignins (Barrière *et al.*, 2004). To evaluate the functionality of the mutated BdCOMT6 protein (BdCOMT6^5139^), complementation experiments were performed in an *Arabidopsis comt-1* mutant which is almost completely depleted in S lignin units (Vanholme *et al.*, 2012; Van Acker *et al.*, 2013). Such a complementation experiment was performed in order to determine to what extent the *BdCOMT6* ^*5139*^ allele was able to rescue the biosynthesis of syringyl lignin units in the *Arabidopsis comt-1* mutant.

**Fig. 3.**
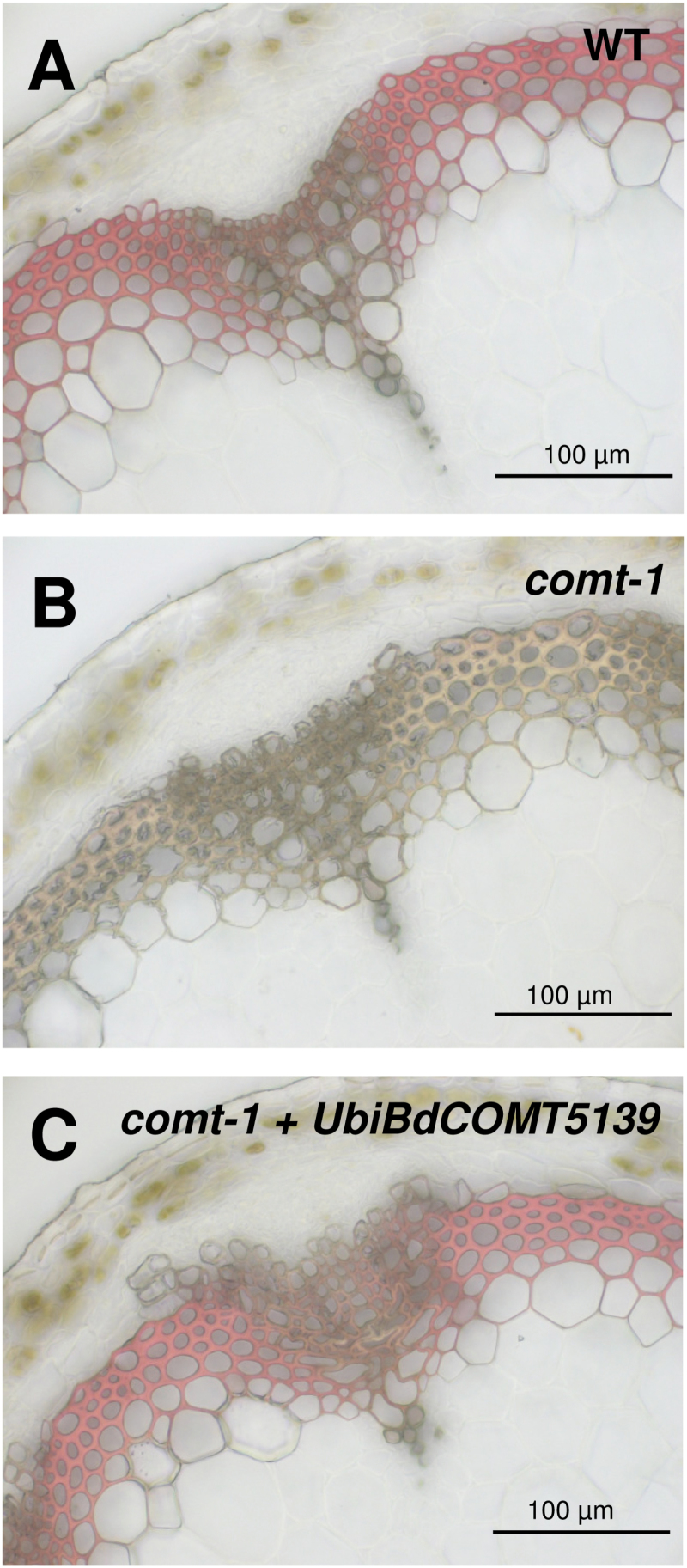
*Arabidopsis comt-1* complementation assays using mutated *BdCOMT6.* Stem cross-sections of *Arabidopsis thaliana* stained using the Maüle reagent to reveal S lignin units. (A) WT (Col-0) with intense positive staining in lignified interfascicular fibres, (B) *comt-1* mutant depleted in S lignin units (negative Maüle staining), (C) *comt-1* line complemented with the mutated *BdCOMT6* gene under the control of the maize ubiquitin promoter (restoration of positive Maüle staining). Scale bar=100 μm (A–C). (This figure is available in colour at *JXB* online.)

Not unexpectedly, the Maüle reagent specific for S lignin units negatively stained *Arabidopsis* stem cross-sections from the *comt-1* line (Fig. 3). A positive Maüle staining was restored in *Arabidopsis comt-1* samples complemented with the *BdCOMT6* ^*5139*^ allele (Fig. 3). This result revealed that the BdCOMT6^5139^ protein has conserved sufficient activity to catalyse the methylation step involved in the pathway to sinapyl alcohol. To evaluate the functionality of the mutated protein more precisely, the impact of the complementation on the frequency of lignin-derived H, G, S, and 5-OH G thioacidolysis monomers specifically released from the lignins of mature *Arabidopsis* stems was examined (Table 4). As expected and relative to the WT levels, the relative frequency of the 5-OH G monomers was dramatically increased while that of S monomers was reduced to <3% in the *comt-1 Arabidopsis* mutant. The transformation of this mutant with *BdCOMT6* ^*5139*^ rescued the S lignin units to a frequency that surprisingly exceeded that of the WT sample and for three different complemented lines (Table 4). In addition, the frequency of 5-OH G thioacidolysis monomers was decreased to a value close to that of the WT. These thioacidolysis results unambiguously confirmed that the mutated BdCOMT6 has conserved sufficient enzyme activity so as to rescue the COMT-deficient *Arabidopsis* mutant efficiently. The surprisingly higher frequency of S units in the complemented lines and relative to the control value might be accounted for by the promoter employed, the strong maize ubiquitin promoter, which would lead to a non-specific overexpression of the mutated *BdCOMT6* ^*5139*^ gene as compared with the regulated WT *AtCOMT1* transcript.

**Table 4.** *Relative molar frequency of the* p*-hydroxyphenyl (H), guaiacyl (G), syringyl (S), and 5-hydroxyguaiacyl (5-OH G) monomers released by thioacidolysis of the cell wall residues from* Arabidopsis *mature stems* The examined genotypes (in the Col-0 background) are the wild type (WT), the *comt-1* mutant, and three lines (Cp-line) obtained by complementation of the *comt-1* mutant with the mutated *BdCOMT6* gene.

Genotype	%H	%G	%S	% 5-OH G
WT	0.83±0.02a	69.3±0.2 a	29.3±0.4a	0.51±0.02 a
*comt-1*	0.84±0.04 a	90.4±0.6 b	2.40±0.69 b	6.34±0.09 b
Cp line 4–7	1.63±0.10 b	59.6±0.2 c	38.2±0.3 c	0.59±0.01 a
Cp line 7-5	1.74±0.19 b	59.7±0.3 c	38.0±0.2 c	0.60±0.02 a
Cp line 15–6	1.57±0.12 b	58.7±0.5 c	39.1±0.4 c	0.61±0.03 a

Values are means ±SD (with three biological replicates).

Within each row, different letters indicate significant differences (one-way ANOVA) at *P*<0.01.

In previous studies, it has been shown that an *Arabidopsis* T-DNA mutant knockout for the lignin-specific *comt1* gene was not only affected in stem lignification, but also in the pool of soluble phenolics (Goujon *et al.*, 2003; Do *et al.*, 2007). This mutant contained a severely reduced level of sinapoyl malate (SIM) and abnormal amounts of 5-hydroxyferuloyl malate (5-OH FM). In addition and at the plantlet stage, *AtCOMT1* deficiency induced the complete disappearance of isorhamnetin glycosides. These results established that the AtCOMT1 enzyme is involved not only in the lignin pathway, but also in the synthesis of SIM and of methylated flavonol derivatives (i.e. isorhamnetin derivatives). To establish further the functionality of the mutated BdCOMT6 protein, the impact of the complementation experiment on the soluble phenolics of 5-day-old *comt-1 Arabidopsis* plantlets was investigated. In agreement with previous results (Do *et al.*, 2007) and relative to the WT values, the *comt-1* plantlets contained a severely reduced SIM level, this reduction being partially compensated for by the appearance of 5-OH FM (Supplementary Table S2 at *JXB* online). In addition, isorhamnetin derivatives that could be observed as minor soluble phenolics of WT plantlets were entirely absent from *comt-1* plantlets. The complementation with the mutated BdCOMT6 protein efficiently restored the SIM and the isorhamnetin levels to the WT value and induced the total disappearance of the 5-OH FM.

Taken together, these results ascertained that the mutated *BdCOMT6* gene introduced in the *AtCOMT1*-deficient *Arabidopsis* mutant was able to rescue not only its altered stem lignification, but also its reduced pool of methylated soluble phenolics. A similar rescue of syringyl units and of sinapate esters has been reported for the *fah* Arabidopsis mutant complemented by the *Eucalyptus globulus* coniferaldehyde-5 hydroxylase (Garcia *et al.*, 2014). In the present study, such an efficient complementation was done with the mutated *BdCOMT6* gene, which definitely established that the mutated BdCOMT6 protein is still functional.

### The *Bd5139* mutant displays an improved saccharification of mature stems without major alteration in grain quality

A promising breeding strategy of cereal crops would consist of making their straw more amenable to saccharification, through appropriate changes in lignin content and/or structure, without introducing deleterious effects on biomass production and on grain quality. Indeed, as lignins are essential to plant health and development, substantial lignin reduction would inevitably reduce the agricultural fitness of grass crops (Pedersen *et al.*, 2005).

Many studies of COMT-deficient transgenic or mutant angiosperms have established that their reduced lignin level was associated with improved digestibility (Cherney *et al.*, 1991; Bernard-Vailhé *et al.*, 1996; Sewalt *et al.*, 1997; Goujon *et al.*, 2003; Barrière *et al.*, 2004; Chen *et al.*, 2004; Trabucco *et al.*, 2013) or saccharification (Chen and Dixon, 2007; Dien *et al.*, 2011; Fu *et al.*, 2011; Jung *et al.*, 2012; Van Acker *et al.*, 2013; Baxter *et al.*, 2014). The currently studied *Bd5139* mutant displayed moderate lignin reduction, as the BdCOMT6 mutated protein, provided with a single point mutation, has retained enough enzyme activity to ensure a lignin level reduced only by 10–15%. On this basis, the impact of the mutation on the saccharification of mature stems was investigated.

A saccharification assay was conducted on small amounts (30mg) of stem CWR without any pre-treatment and using a commercially available cellulase preparation (cellulase Onozuka from *T. viride*, provided with cellulase, hemicellulase, and β-glucosidase activities). The efficiency of cell wall enzymatic hydrolysis was evaluated both as the weight loss induced by the enzyme treatment and as the glucose amount released from the cell walls. Both evaluation methods consistently revealed that the saccharification efficiency of the stem CWR was improved (by ~20%) by the mutation and relative to the WT values (Table 5). Such a result is consistent with the lower lignin level of *Bd5139* stems as lignins detrimentally affect the enzymatic degradation of lignocellulosic biomass.

**Table 5.** *Saccharification assays of wild-type (WT, accession Bd21-3) and* Bd5139 *cell wall residues prepared from mature stem samples* Saccharification efficiency is measured as the weight loss induced by the enzymatic treatment (as a percentage of the initial weight) and by the glucose released from the cell walls.

Line	Weight loss %	Glucose mg g^–1*a*^
WT	23.0±1.5	80.9±3.1
*Bd5139*	27.7±1.0**	97.7±2.5**

Values are means ±SD (*n*=6 with three biological replicates, each one analysed as analytical duplicates).

Asterisks indicate significant differences (*t*-test) compared with the WT value at **P< 0.01.

^*a*^ Expressed as anhydroglucose equivalent.

Lignins are important components of the stem vascular tissues necessary to convey water and nutrients to the developing grain. In addition, lignins occur in several grain outer layers that fulfil protective and nutritive functions towards the developing seed. Lignin mutants with seed defects have been reported. Maize field trials where *bm3* lines were assessed revealed a reduced grain yield that was explained by a lower number of ears per plant and by a lower number of kernels per ear (reviewed in Pedersen *et al.*, 2005). Several *Arabidopsis* mutants altered in lignin polymerization exhibit seed phenotypes such as defects in seed pigmentation, permeability, and germination (Liang *et al.*, 2006) or increased numbers of siliques and enlarged seeds (Wang *et al*., 2014). The *Bd5139* line was assessed to evaluate the effect of moderate changes in lignin content and composition on cereal grains. Grain size and morphology were not affected in the *Bd5139* line (Supplementary Fig. S5 at *JXB* online). No major effect was noticed on grain histology or development (Supplementary Fig. S5). No effect of the *Bd5139* mutation could be evidenced on grain polysaccharide storage compounds (Supplementary Table S3). Therefore, the moderate decrease in grain lignins and CA esters does not seem to affect grain development and polysaccharide composition.

### Conclusion

In this study focused on the *Bd5139 Brachypodium* mutant, it was established that modifying the lignin-related *BdCOMT6* gene induced alterations in grain lignins which nicely mirrored those observed in stem lignins. The accumulation of 5-OH G units, which is the most specific signature for COMT deficiency, was reported here for the first time in the grain of a COMT grass mutant. The single mutation in the BdCOMT6 protein did not completely annihilate its enzyme activity. It induced substantial alterations in lignin structure but only a moderately reduced lignin level. The moderate lignin reduction did not compromise the vegetative and reproductive development of the *Brachypodium* plant model, but facilitated the straw saccharification, opening up the possibility of a sustainable cereal grain production with improved straw end-use potential.

## Supplementary data

Supplementary data are available at JXB online.

Figure S1. Expression profile of *BdCOMT6.*

Figure S2. Phenotype of *Bd5139* compared with WT (Bd21-3) plants.

Figure S3. Partial GC-MS traces of the lignin-derived thioacidolysis monomers (analysed as their silylated derivatives) released from WT and *Bd5139* grain CWR.

Figure S4. Sequence alignment of BdCOMT6 and characterized COMTs showing residues described as essential for COMT activity.

Figure S5. Grain development and grain size are not affected in *Bd5139*.

Table S1. Determination of *p*-coumaric acid and of 5-*O*-*p*-coumaroyl arabinose released by mild acidolysis (dioxane/water 9/1, v/v, containing 0.2M HCl, 50 °C overnight) of wild type (accession Bd21-3) and *Bd5139* cell wall residues prepared from mature stem samples.

Table S2. LC-MS determination of sinapoyl malate, 5-hydroxy feruloyl malate, and isorhamnetin-3-*O*-glucoside-7-*O*-rhamnoside extracted from 6-day-old *Arabidopsis* plantlets.

Table S3. Sugar composition of the alcohol-insoluble residues prepared from whole grain of wild-type (accession Bd21-3) and *Bd5139* lines.

Supplementary Data

## Abbreviations:

ABL: acetyl bromide lignin
AIR: alcohol-insoluble residue
Ara: arabinose
Bd: *Brachypodium distachyon*
CA: *p*-coumaric acid
COMT: caffeic acid *O*-methyl transferase
CWR: cell wall residue
DAF: days after flowering
DAG: days after germination
FA: ferulic acid
G: guaiacyl
H: *p*-hydroxyphenyl
KL: Klason lignin
S: syringyl
5-OH G: 5-hydroxyguaiacyl.
