# Supplementary material for: Mutation in Brachypodium caffeic acid O-methyltransferase 6 alters stem and grain lignins and improves straw saccharification without deteriorating grain quality
Source: J Exp Bot. 2015 Oct 3;67(1):227–37. doi: 10.1093/jxb/erv446 (PMC4682429; doi:10.1093/jxb/erv446)
Supplement: Supplementary Data [file supp_67_1_227__index.html]

Mutation in Brachypodium caffeic acid O-methyltransferase 6 alters stem and grain lignins and improves straw saccharification without deteriorating grain quality — Mutation in Brachypodium caffeic acid O-methyltransferase 6 alters stem and grain lignins and improves straw saccharification without deteriorating grain quality — Supplementary Data 

# Mutation in *Brachypodium* caffeic acid *O*-methyltransferase 6 alters stem and grain lignins and improves straw saccharification without deteriorating grain quality

## Supplementary Data

Data files

- Supplementary Data - Supplementary Data
