# Supplementary material for: Mutation in Brachypodium caffeic acid O-methyltransferase 6 alters stem and grain lignins and improves straw saccharification without deteriorating grain quality
Source: J Exp Bot. 2015 Oct 3;67(1):227–37. doi: 10.1093/jxb/erv446 (PMC4682429; doi:10.1093/jxb/erv446)
Supplement: Supplementary Data [file supp_erv446_jexbot149963_file001.pdf]

Mutation in Brachypodium caffeic acid O-methyl transferase 6 alters stem and grain lignins and improves straw saccharification without deteriorating grain quality.

Séverine Ho-Yue-Kuang, Camille Alvarado, Sebastien Antelme, Brigitte Bouchet, Laurent. Cezard, Philippe Le Bris, Frédéric Legée, Alessandra Maia-Grondard, Arata Yoshinaga, Luc Saulnier, Fabienne Guillon, Richard Sibout, Catherine Lapierre, and Anne-Laure Chateigner-Boutin

Supplemental Files

Supplementary Fig. S1. Expression profile of *BdCOMT6*.

A

Y axis Log2(signal value)

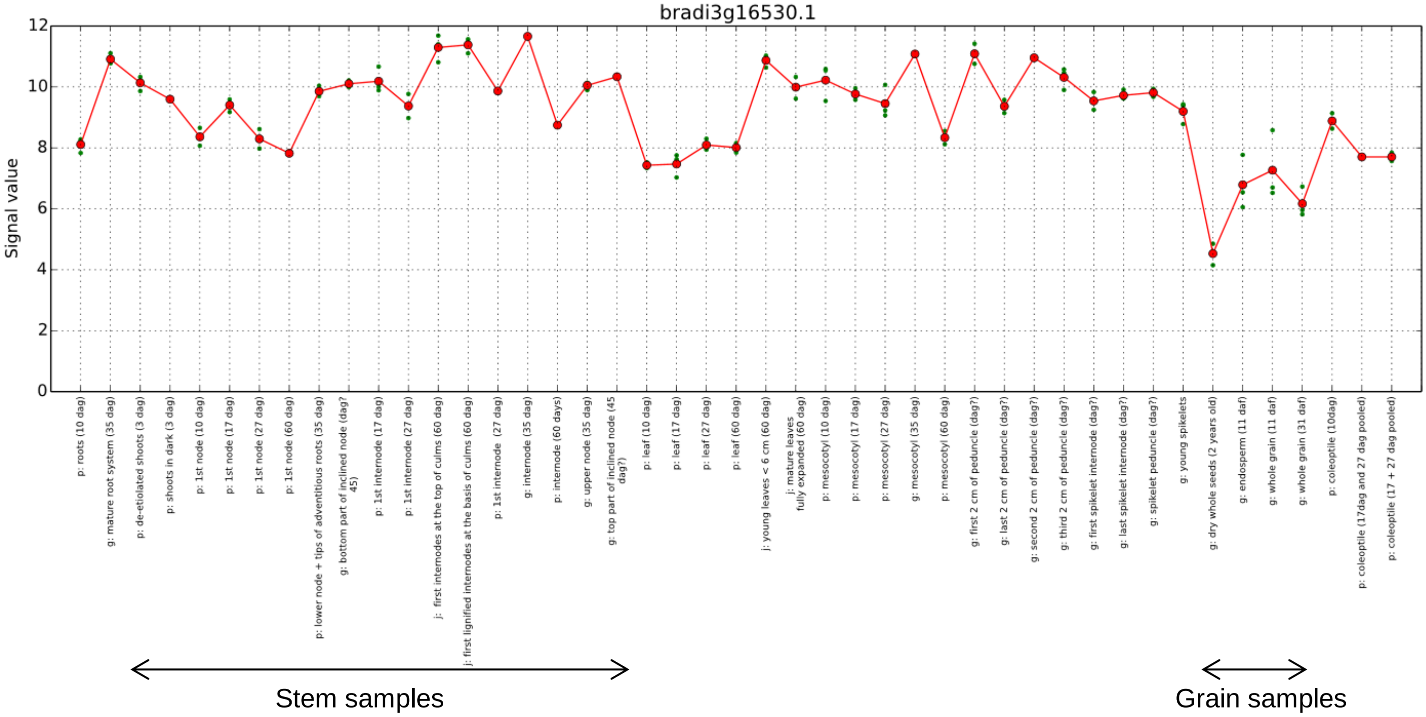

Expression profile obtained from the Planet Expression platform : http://aranet.mpimp-golm.mpg.de/index.html based on Affymetrix microarray experiments.

B

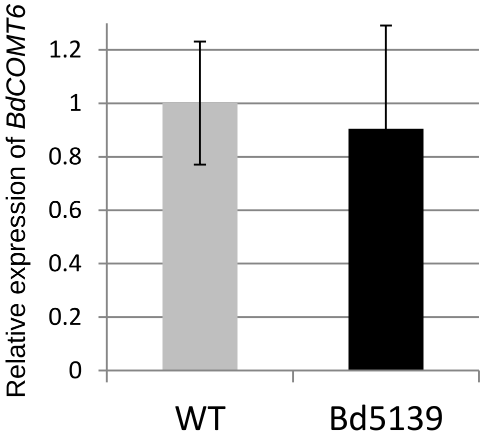

Comparison of the expression of *BdCOMT6* in stems of Bd21-3 (WT) and Bd5139 60 DAG plants by qRT-PCR. Transcript levels of *BdCOMT6* were normalized against the housekeeping gene S-adenosylmethionine decarboxylase (SamDC) and the expression value was set to 1 for WT (Experimental procedures detailed in Bouvier d'Yvoire et al., 2013; BDCOMT-6-F GTTCCACGTCGACATGATCAT, BDCOMT-6-R CTACTTGGTGAACTCGATGGC, SAMDC-F CGGCAAGCTTGCTAATCTGCTGGAAT, SAMDC-R CAGAGCAACAATAGCCTGGCTGGC). Error bars are SD of three biological replicates.

Supplementary Fig. S2 : Phenotype of Bd5139 compared to WT(Bd21-3) plants.

A

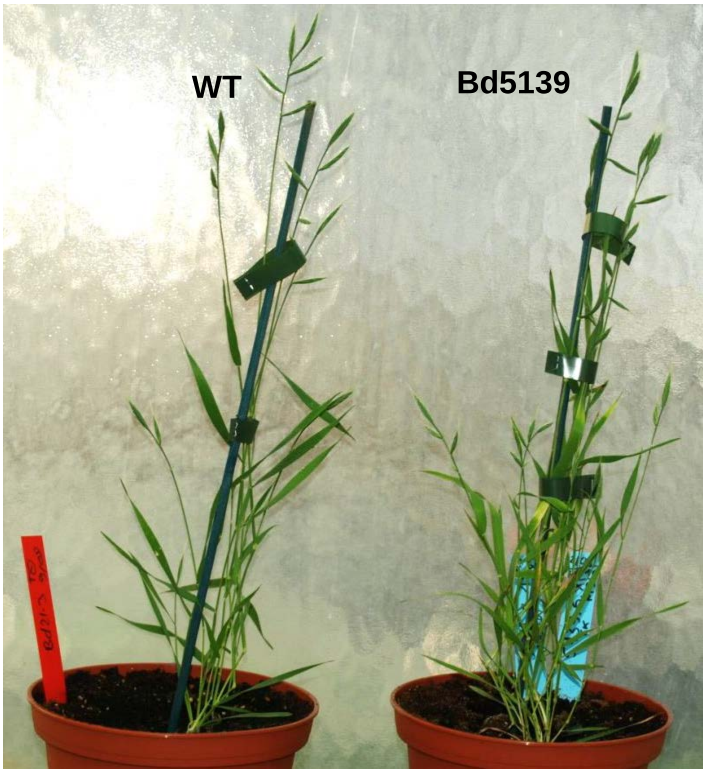

Bd5139 plants cultivated in a growth chamber for 60 days do not show any obvious phenotype when compared to WT plants grown in the same chamber at the same time .

B

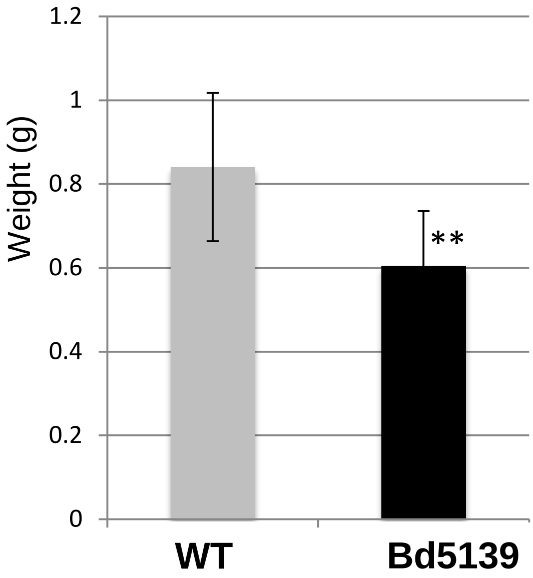

Biomass comparison between 8 WT (Bd21-3) and 8 Bd5139 plants grown in the same chamber at the same time. Grains and leaves were removed from harvested dry plants and stems were weighted. A slight decrease in biomass was observed. Bars : SD; **p<0,01(t-test).

Supplementary Fig. S3: Thioacidolysis monomers from Brachypodium grain.

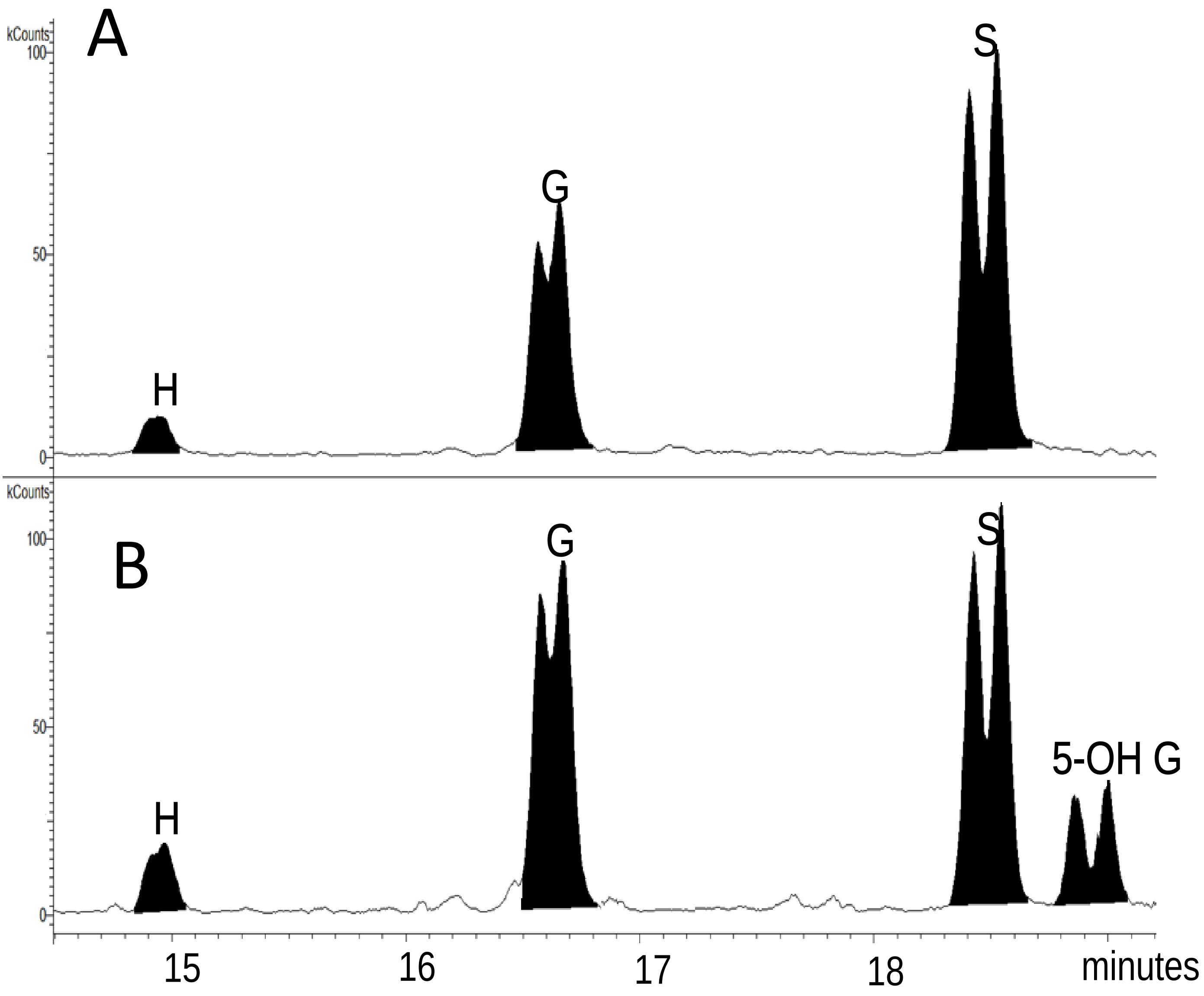

Partial GC-MS traces showing the separation of the *p*-hydroxyphenyl H, guaiacyl G, syringyl S and 5-hydroxyguaiacyl 5-OH G monomers released by thioacidolysis of cell wall residues from A) Wild-type whole grains and B) Bd5139 whole grains. These monomers are analyzed as their trimethylsilylated derivatives and are otained as a mixture of erythro/threo diastereoisomers

Supplementary Fig. S4 : Sequence alignment of BdCOMT6 and characterized COMTs showing residues described as essential for COMT activity (performed with CLUSTAL 2.1).

Highlighted : residues involved dimerisation (green), catalytic activity (yellow), interaction with cofactor (purple) , interaction with substrate (blue). Lp : *Lolium perenne*, Ms: *Medicago sativa*, Sb: *Sorghum bicolor*, Zm: *Zea mays*, At: *Arabidopsis thaliana*. The amino acid mutated in Bd5139 is shown by a red arrow.

```
Lp          MGSTAADMAA---SADEDACMFALQLASSSVLPMTLKNAIELGLLEILVAAGG---KSLT 54
BdCOMT6     MGSTAADMAA---TADEEACMFALQLASSSILPMTLKNAIELGLLDTLVQASG---KSLT 54
Zm          MGSTAGDVAA---VVDEEACMYAMQLASSSILPMTLKNAIELGLLEVLQKEAGGGKAALA 57
Sb          MGSTAEDVAA---VADEEACMYAMQLASSSILPMTLKNALELGLLEVLQKDAG---KALA 54
Ms          MGSTGETQITPTHISDEEANLFAMQLASASVLPMILKSALELDLLEIIAKAGP--GAQIS 58
At          MGSTAETQLTPVQVTDDEAALFAMQLASASVLPMALKSALELDLLEIMAKN----GSPMS 56
            ****.    :     *::* ::*:****:*:*** **.*:**.**: :         ::

Lp          PTEVAAKLP-SAANP-EAPDMVDRILRLLASYNVVTCLVEEGKDGRLSRSYGAAPVCKFL 112
BdCOMT6     PAEVAAKLP-SSSNP-AAPDMVDRMLRLLASYGVVSCAVEEGENGKLSRRYAAAPVCKWL 112
Zm          PEEVVARMPAAPSDPAAAAAMVDRMLRLLASYDVVRCQMED-RDGRYERRYSAAPVCKWL 116
Sb          AEEVVARLPVAPTNP-AAADMVDRMLRLLASYDVVRCQMED-KDGKYERRYSAAPVGKWL 112
Ms          PIEIASQLP--TTNP-DAPVMLDRMLRLLACYIILTCSVRTQQDGKVQRLYGLATVAKYL 115
At          PTEIASKLP--TKNP-EAPVMLDRILRLLTSYSVLTCSNRKLSGDGVERIYGLGPVCKYL 113
            . *:.:::*   . :*  *. *:**:****:.* :: *  .   ..  .* *. ..* *:*

Lp          TPNEDGVSMAALALMNQDKVLMESWYYLKDAVLDGGIPFNKAYGMSAFEYHGTDPRFNRV 172
BdCOMT6     TPNEDGVSMAALALMNQDKVLMESWYYLKDAVLDGGIPFNKAYGMSAFEYHGTDPRFNRV 172
Zm          TPNEDGVSMAALALMNQDKVLMESWYYLKDAVLDGGIPFNKAYGMTAFEYHGTDARFNRV 176
Sb          TPNEDGVSMAALALMNQDKVLMESWYYLKDAVLDGGIPFNKAYGMTAFEYHGTDPRFNRV 172
Ms          VKNEDGVSISALNLMNQDKVLMESWYHLKDAVLDGGIPFNKAYGMTAFEYHGTDPRFNKV 175
At          TKNEDGVSIAALCLMNQDKVLMESWYHLKDAILDGGIPFNKAYGMSAFEYHGTDPRFNKV 173
            . ******::** *************:****:*************:********.***:*

Lp          FNEGMKNHSIIITKKLLELYHGFEG-LGTLVDVGGGVGATVAAIAAHYPTIKGVNFDLPH 231
BdCOMT6     FNEGMKNHSIIITKKLLDLYPGFEG-LGTLVDVGGGVGATVGAIVARHPAIKGINFDLPH 231
Zm          FNEGMKNHSVIITKKLLDFYTGFEG-VSTLVDVGGGVGATLHAITSRHPHISGVNFDLPH 235
Sb          FNEGMKNHSVIITKKLLEFYTGFDESVSTLVDVGGGIGATLHAITSHHSHIRGINFDLPH 232
Ms          FNKGMSDHSTITMKKILETYTGFEG-LKSLVDVGGGTGAVINTIVSKYPTIKGINFDLPH 234
At          FNNGMSNHSTITMKKILETYKGFEG-LTSLVDVGGGIGATLKMIVSKYPNLKGINFDLPH 232
            **:**.:** *  **:*: * **:   : :******* **.:   *.:::. : *:******

Lp          VISEAPQFPGVTHVGGDMFKEVPSGDAILMKWILHDWSDQHCATLLKNCYDALPAHG-KV 290
BdCOMT6     VISEGIPFPGVTHVGGDMFQKVPSGDAILMKWILHDWSDAHCATLLKNCYDALPAHG-KV 290
Zm          VISEAPPFPGVRHVGGDMFASVPAGDAILMKWILHDWSDAHCATLLKNCYDALPENG-KV 294
Sb          VISEAPPFPGVQHVGGDMFKSVPAGDAILMKWILHDWSDAHCATLLKNCYDALPEKGGKV 292
Ms          VIEDAPSYPGVEHVGGDMFVSIPKADAVFMKWICHDWSDEHCLKFLKNCYEALPDNG-KV 293
At          VIEDAPSHPGIEHVGGDMFVSVPKGDAIFMKWICHDWSDEHCVKFLKNCYESLPEDG-KV 291
            **.:.   .**: ******* .:* .*:::**** ***** ** .:*****::** .* **

Lp          VLVQCILPVNPEANPSSQGVFHVDMIMLAHNPGGRERYEREFQALARGAGFTGVKSTYIY 350
BdCOMT6     VIVECILPVNPEATPKAQGVFHVDMIMLAHNPGGKERYEREFEELARGAGFTGVKATYIY 350
Zm          IVVECVLPVNTEATPKAQGVFHVDMIMLAHNPGGKERYEREFRELAKGAGFSGFKATYIY 354
Sb          IVVECVLPVTTDAVPKAQGVFHVDMIMLAHNPGGRERYEREFRDLAKAAGFSGFKATYIY 352
Ms          IVAECILPVAPDSSLATKGVVHIDVIMLAHNPGGKERTQKEFEDLAKGAGFQGFKVHCNA 353
At          ILAECILPETPDSSLSTKQVVHVDCIMLAHNPGGKERTEKEFEALAKASGFKGIKVVCDA 351
            ::.:*:**  .::   :: *.*:* *********:** ::**. **:.:** *.*

Lp          ANAWAIEFTK-- 360
BdCOMT6     ANAWAIEFTK-- 360
Zm          ANAWAIEFIK-- 364
Sb          ANAWAIEFIK-- 362
Ms          FNTYIMEFLKKV 365
At          FGVNLIELLKKL 363
             ..   :*: *
```

* (asterisk) indicates positions which have a single, fully conserved residue.
: (colon) indicates conservation between groups of strongly similar properties
. (period) indicates conservation between groups of weakly similar properties

Supplementary Fig. S5. Grain development and grain size are not affected in Bd5139.

Top : differential interference contrast images of sections of grains harvested at different stages of development.
Bottom : images of WT and Bd5139 grains.

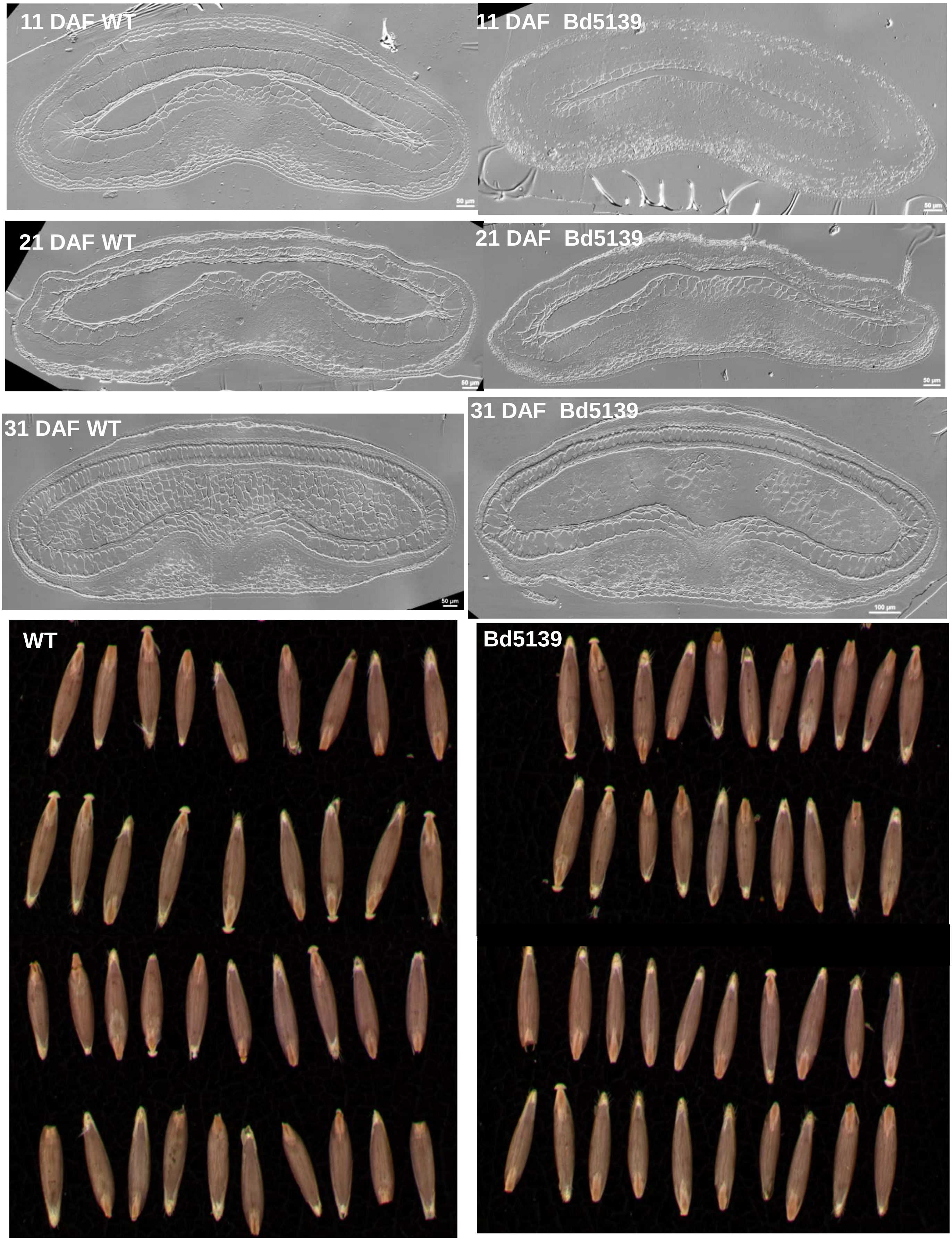

Supplementary Table S1. Determination of *p*-coumaric acid (CA) and of 5-*O*-*p* coumaroyl arabinose (CA-Ara) released by mild acidolysis (dioxane/water 9/1, v/v, containing 0.2M HCl, 50°C overnight) of wild type (WT, accession Bd21-3) and *Bd5139* cell wall residues (CWR) prepared from mature stem samples.

Line	CA mg/g	CA-Ara mg/g
WT	0.85 ± 0.10	3.88 ± 0.38
Bd5139	0.61 ± 0.10	3.58 ± 0.15

Values are means ± SD (n=3).

Supplementary Table S2. LC-MS determination of sinapoyl malate (SIM), 5-hydroxy feruloyl malate (5-OH FM) and isorhamnetin-3-*O*-glucoside-7-*O*-rhamnoside (IGR) extracted from 6-day old Arabidopsis plantlets. The examined Arabidopsis genotypes are the wild-type (WT) Col0 one, the *comt-1* mutant and three lines (Cp-line) obtained by complementation of the comt-1 mutant with the mutated *BdCOMT6* gene. The results are expressed in mg/g fresh weight.

Genotype	SIM mg/g	5-OH FM mg/g	IGR mg/g
WT Col0	2.60 ± 0.31	ND	0.14 ± 0.03
comt-1	1.09 ± 0.17*	0.43 ± 0.10	ND
Cp line 4-7	2.11 ± 0.13	ND	0.06 ± 0.01*
Cp line 7-5	1.93 ± 0.28	ND	0.07 ± 0.01*
Cp line 15-6	2.73 ± 0.22	ND	0.09 ± 0.01

Values are means ± SD from biological triplicates, each assay being conducted from about 20 plantlets. ND : not detectable. Asterisks indicate significant differences (one way ANOVA) compared to the WT value at $p < 0.01$

1

Supplementary Table S3. Sugar composition of the alcohol insoluble residues prepared from whole grain of wild type (WT, accession Bd21-3) WT and *Bd5139* lines. Arabinose (Ara), xylose (Xyl), glucose (Glu), mannose (Man) and galactose (Gal) content was determined by gas-liquid chromatography of alditol acetates.

Line	Neutral sugars					
	Total	Ara	Xyl	Glu	Man	Gal
WT	62.6	2.9±0.3	5.1±0.2	53.9±0.9	0.2±0.03	0.6±0.02
Bd5139	61.3	2.7±0.1	4.6±0.2	53.3±1.0	0.15±0.0	0.6±0.02

Whole grain samples correspond to the whole caryopsis with the adhering palea. They were prepared from 70 grains collected from at least 30 plants for each genotype. Values are means ± SD from 2 analytical replicates expressed as mg/100 mg of dried alcohol insoluble residues.
